# Peptaibol-Containing Extracts of *Trichoderma atroviride* and the Fight against Resistant Microorganisms and Cancer Cells

**DOI:** 10.3390/molecules26196025

**Published:** 2021-10-04

**Authors:** Ján Víglaš, Simona Dobiasová, Jitka Viktorová, Tomáš Ruml, Vanda Repiská, Petra Olejníková, Helena Gbelcová

**Affiliations:** 1 Institute of Biochemistry and Microbiology, Faculty of Food and Chemical Technology, Slovak University of Technology in Bratislava, 812 37 Bratislava, Slovakia; petra.olejnikova@stuba.sk; 2 Department of Biochemistry and Microbiology, University of Chemistry and Technology Prague, 166 28 Prague, Czech Republic; simka.dobiasova@gmail.com (S.D.); jitka.prokesova@vscht.cz (J.V.); tomas.ruml@vscht.cz (T.R.); 3 Institute of Medical Biology, Genetics and Clinical Genetics, Faculty of Medicine, Comenius University in Bratislava, 813 72 Bratislava, Slovakia; vanda.repiska@fmed.uniba.sk (V.R.); helena.gbelcova@fmed.uniba.sk (H.G.)

**Keywords:** peptaibols, *Trichoderma* spp., antimicrobial peptides, anticancer peptides

## Abstract

Fighting resistance to antibiotics and chemotherapeutics has brought bioactive peptides to the fore. Peptaibols are short α-aminoisobutyric acid-containing peptides produced by *Trichoderma* species. Here, we studied the production of peptaibols by *Trichoderma atroviride* O1 and evaluated their antibacterial and anticancer activity against drug-sensitive and multidrug-resistant bacterium and cancer cell lines. This was substantiated by an analysis of the activity of the peptaibol synthetase-encoding gene. Atroviridins, 20-residue peptaibols were detected using MALDI-TOF mass spectrometry. Gram-positive bacteria were susceptible to peptaibol-containing extracts of *T. atroviride* O1. A synergic effect of extract constituents was possible, and the biolo-gical activity of extracts was pronounced in/after the peak of peptaibol synthetase activity. The growth of methicillin-resistant *Staphylococcus aureus* was reduced to just under 10% compared to the control. The effect of peptaibol-containing extracts was strongly modulated by the lipoteichoic acid and only slightly by the horse blood serum present in the cultivation medium. Peptaibol-containing extracts affected the proliferation of human breast cancer and human ovarian cancer cell lines in a 2D model, including the multidrug-resistant sublines. The peptaibols influenced the size and compactness of the cell lines in a 3D model. Our findings indicate the molecular basis of peptaibol production in *T. atroviride* O1 and the potential of its peptaibol-containing extracts as antimicrobial/anticancer agents.

## 1. Introduction

Antimicrobial peptides (AMPs) and anticancer peptides (ACPs) are the subjects of a search for new therapeutic agents capable of combating the growing resistance to commonly used antimicrobial compounds (microorganisms) and anticancer drugs (cancer cells) [1,2]. The advantage of AMPs over antibiotics is the mechanism of action. AMPs act generally in two ways: (*i*) they disrupt the cytoplasmic membrane by membrane thinning or formation of pores (e.g., toroidal pores in the case of cationic antimicrobial peptides) or (*ii*) are translocated into cytoplasm and bind to different targets (and inhibit nucleic acid synthesis, protein synthesis, cell wall synthesis, etc.) [3]. The described diverse modes of action represent an obstacle for the pathogen in terms of evolving an effective mechanism of resistance due to the lack of a specific target for an antimicrobial compound [3,4]. However, there are exceptions, which involve the incorporation of positively charged molecules into the cell surface to reduce the interaction with cationic AMPs. This is the case for colistin resistant *Escherichia coli* [5,6]. Cationic ACPs have a similar mode of action. Cancer cells membranes are enriched in anionic molecules compared to normal mammalian membranes. These molecules include phosphatidylserine, *O*-glycosylated mucins, sia-lylated gangliosides, and heparin sulfate, giving the membrane a negative net charge, i.e., increasing the selectivity for cationic ACP targeting cancer cells [7].

*Trichoderma* (teleomorph *Hypocrea*) is a fungal genus involving species acting as biocontrol agents (due to a mycoparasitic lifestyle on phytopathogens and promotion of plant growth) that are also highly potential producers of industrially important enzymes [8,9]. *Trichoderma* sp. are producers of a plethora of secondary metabolites, including peptaibiotics, 4–21 residue peptides (500–2100 Da) with α-aminoisobutyric acid (Aib) and other noncanonical amino acids frequently occurring in the main peptide chain. In the largest subgroup, peptaibols, acetylated N-terminus, and amide-bound amino alcohol at the C-terminus are also present. These secondary metabolites are synthesized by peptaibol synthetases, which belong to non-ribosomal peptide synthetases (NRPSs) [10,11]. NRPSs also occur in bacteria, where they synthesize important antibiotics such as colistin and vancomycin [12]. Peptaibols of *Trichoderma* sp. participate in mycoparasitic interaction with phytopathogens as well as in communication with plants (stimulation of plant resistance against phytopathogens) [13,14,15]. The peptaibols are synthetized as a mixture of peptides with variation in some positions in the nascent chain [16,17]. The most studied are 20-residue peptaibols called alamethicins, specifically one member “alamethicin” Ac-Aib-Pro-Aib-Ala-Aib-Ala-Gln-Aib-Val-Aib-Gly-Leu-Aib-Pro-Val-Aib-Aib-Glu-Gln-Phol, which is commercially available (Sigma-Aldrich, St. Louis, MO, USA). Alamethicin serves as a model for the study of the interaction of neutrally charged peptides with plasma membranes. Once inserted in the membrane, alamethicin is organized perpendicularly to the phospholipid bilayer, with hydrophobic side chains of amino acids interacting with membrane lipids. Eight molecules can create a pore (called a barrel-stave pore) allowing plasma membrane depolarization and permeabilization through the water channel of the pore [18]. Structurally, alamethicin is a helical peptide [19]. The antibacterial activity of alamethicin has been described on Gram-positive bacteria and fungi [16]. A variety of new sequences has been described over the past few decades, in some instances even with basic screening for antimicrobial activity [16,20,21,22]. Genetic studies of peptaibol synthetases are restricted to the study of homologues of Tex1 peptaibol synthetase of *Trichoderma virens*, 2.3-MDa NRPSs [15,23,24].

Peptaibols, including alamethicin, are also being studied for anticancer activity [16,25]. The peptaibol trichokonin VI of *Trichoderma pseudokoningii* inhibits metabolic activity of hepatocellular carcinoma cells in a dose-dependent manner, while it does not impair the viability of normal liver cells at a lower concentration [26]. The antimicrobial and cytotoxic activity of alamethicin has been reviewed by Leitgeb et al., (2007) [16]. However, since then, the phenomenon of resistance to commonly used antibiotics and chemotherapeutics has become even more alarming. Therefore, the revision of even known peptaibols for their biological activity has gained relevance.

This study aimed to evaluate the potential of peptaibols-containing extracts obtained from the mycelium of *Trichoderma atroviride* O1 strain in the context of the fight against resistance, a phenomenon with increasing occurrence, from bacteria to cancer cells. We detected peptaibols typical for *T. atroviride*, and biological activity was shown, where multi-drug resistant bacterial strain and cancer cell sub-lines seemed to be most affected. The inhibitory activity of peptaibol-containing extracts was supported at a molecular level by the expression pattern of the peptaibol synthetase encoding gene *tex1_Ta* (homologous to *tex1* of *T. virens*). The nature of peptaibol extracts sets this study in the field of synergic approaches (the peptaibols are isolated as a mixture acting together, and other components of extracts may also contribute).

## 2. Results

### 2.1. Identification of Peptaibols Production from T. atroviride O1

**Figure 1:**
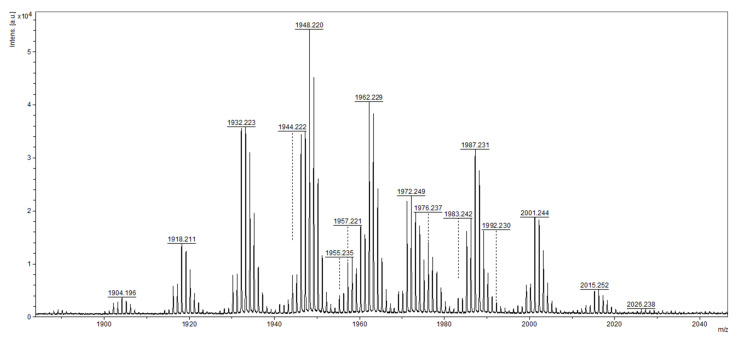
MALDI-TOF mass spectrum profile of crude extract of peptaibols on the 12th day of cultivation in light–dark conditions. The *m/z* values correspond to the atroviridins group of peptaibols, as described by Komon-Zelazowska et al., (2007) [28].

**Figure 2:**
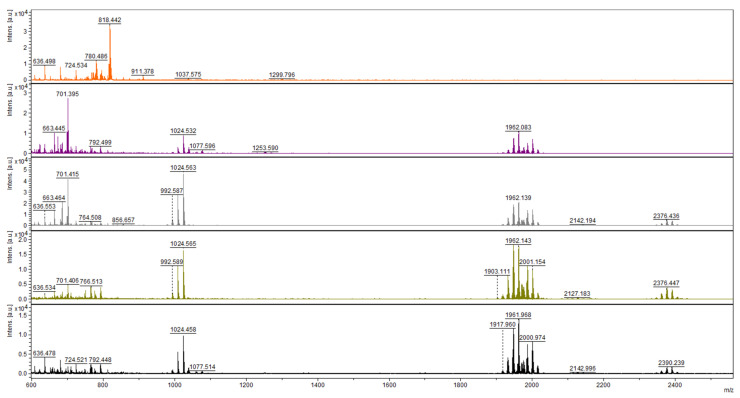
Mass spectra of short peptides isolated from the culture of *T.* *atroviride* O1 cultivated in light–dark conditions (organized in rows). Day of cultivation from the top—2nd, 3rd, 5th, 6th, and 12th. Atroviridins spectrum (*m*/*z* between 1900 and 2000) was first detected on the 3rd day of cultivation (second row).

**Figure 3:**
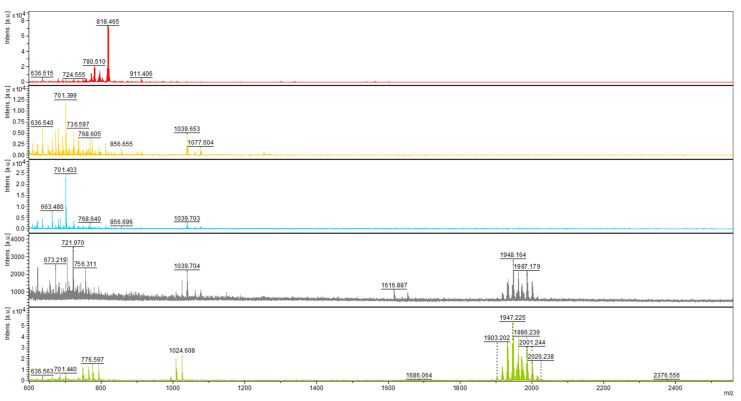
Mass spectra of short peptides isolated from the culture of *T. atroviride* O1 cultivated in constant darkness (organized in rows). Day of cultivation from the top—2nd, 3rd, 5th, 6th, and 12th. Atroviridins spectrum (*m*/*z* between 1900 and 2000) was first detected on the 6th day of cultivation (fourth row).

The presence of peptaibols in crude extracts was monitored by MALDI-TOF mass spectrometry. First, by analyzing the extracts from the 12th day of cultivation (regardless of light regime), we were able to detect peaks in the range of *m/z* values from 1904 to 2015 (Figure 1), as had been achieved previously [27]. The range was practically identical, with the mass spectrum of atroviridins detected in *Hypocrea atroviridis* (teleomorph = sexual reproductive stage of *Trichoderma atroviride*) [28], which led us to the conclusion that *T. atroviride* O1 produces atroviridins. MS/MS studies were also applied using collision ion dissociation through a MALDI-TOF/TOF mass spectrometer on chosen peaks in the range corresponding to peptaibols of *T. atroviride*, where we detected α-aminoisobutyric acid, typical for peptaibols (Figures S1–S3, Supplementary Materials). Analysis of crude extracts of peptaibols collected at individual time points of cultivation revealed that in the light–dark conditions the peaks corresponding to atroviridins were detectable from the third day of cultivation (Figure 2), while in the darkness the beginning of peptaibol production was on the sixth day (Figure 3). These metabolites were detectable until the end of cultivation, indicating the accumulation of peptaibols, a result of active peptaibol synthetase encoded by the gene *tex1_Ta*, also called *pbs1* [28]. MALDI-TOF analysis also revealed other molecules (Figure 2 and Figure 3) detected throughout the cultivation, namely peaks around *m/z* 800, 1000 and 2140, and 2370–2390. These constituents of peptaibols-containing extracts may represent less known peptaibols or other natural products, simply compounds of a probable peptide nature (extraction method designed for peptaibols = peptides), which may contribute to the synergic effect of peptaibols, and perhaps other effects are also possible on cancer cell lines (see Discussion—first and last paragraph).

### 2.2. Expression Profile of the Gene tex1_Ta Encoding Peptaibol Synthetase

**Figure 4:**
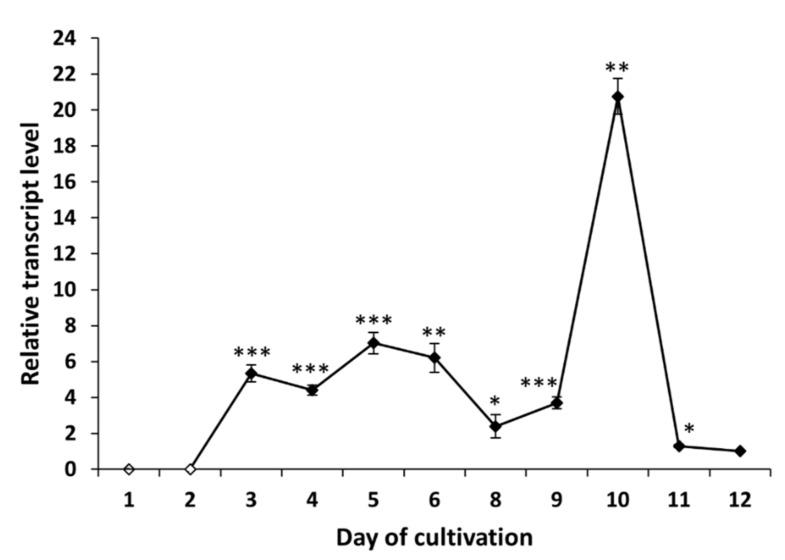
Expression profile of peptaibol synthetase *tex1_Ta Trichoderma atroviride* O1 cultivated in light–dark conditions (LD). White points indicate undetected *tex1_Ta* transcript. Reference (relative transcript level = 1) is the expression level detected on the 12th day of cultivation (12 d LD). Values with *p* < 0.001, 0.001 < *p* < 0.01, and 0.01 < *p* < 0.05 (compared to reference) are marked with ***, ** and *, respectively.

**Figure 5:**
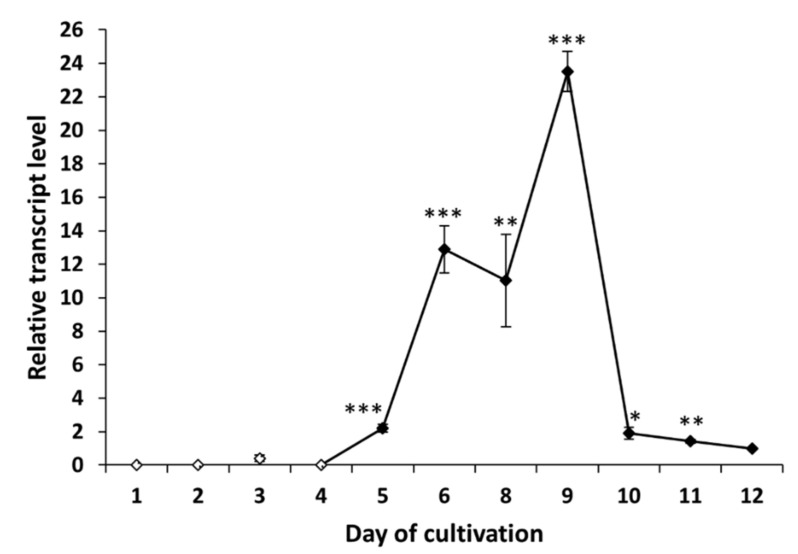
Expression profile of peptaibol synthetase *tex1_Ta Trichoderma atroviride* O1 cultivated in constant darkness (D). White points indicate undetected *tex1_Ta* transcript or, on the third day of cultivation, an insignificant amount of transcript. Reference (relative transcript level = 1) is the expression level detected on the 12th day of cultivation (12 d D). Values with *p* < 0.001, 0.001 < *p* < 0.01, and 0.01 < *p* < 0.05 (compared to reference) are marked with ***, ** and *, respectively.

The gene *tex1_Ta* encodes a 19-module peptaibol synthetase (21,879 amino acids) that is responsible for the formation of atroviridins [28]. The transcript analysis of *tex1_Ta T. atroviride* O1 revealed changes in the expression of peptaibol synthetase over time. In the case of cultivation in light–dark conditions, we detected the transcript of the *tex1_Ta* gene on the third day of cultivation (Figure 4), which was the same day the presence of peptaibols was verified in peptaibol-containing extracts (Figure 2). The relative amount of the transcript on the third day of cultivation in light–dark conditions was increased more than 5-fold compared to the reference value (expression on the 12th day - chosen due to a low yet detectable transcript level, regardless of light conditions of cultivation; the peak of *tex1_Ta* expression is best visualized), and the transcript levels fluctuated around this value up to the ninth day. The peak of expression was detected on the tenth day, i.e., late stage of the cultivation, followed by a steep decline on the 11th day to a level comparable with the reference. Conversely, the expression pattern of *tex1_Ta* was different when *T. atroviride* O1 was cultivated in constant darkness (Figure 5). There was practically no transcript detected for the first four days. A transcript level 2-fold higher than the reference was detected on the fifth day. At this stage, the MALDI-TOF spectrum of the extract did not show peptaibol peaks in the *m/z* range of 1900–2000 (Figure 3). The expression of *tex1_Ta* grew rapidly, reaching its peak on the ninth day of cultivation and, again, followed by a reduction in expression on the tenth day to the level just under 2-fold compared with the reference (12th day). As we show in Section 2.3 and Section 2.4, the inhibitory activity of peptaibols-containing extracts appeared correlated with peptaibol synthetase expression (in/after the peak of expression), what suggested after sufficient production of peptaibols (and possibly other metabolites, allowing them to show their synergic effect, though other effects may occur by cytotoxic activity as well).

### 2.3. Antibacterial Activity of Peptaibols T. atroviride O1

The extracts of atroviridins from *T. atroviride* O1 were found to have an inhibitory activity against Gram-positive bacteria (Table 1). Intriguingly, the effect was more pronounced on MRSA (methicillin-resistant *S. aureus*—clinical isolate) than on the collection strain *S. aureus* CCM3953. The inhibition efficiency depended on the applied extract. The peptaibol-containing extracts from *T. atroviride* O1 cultivated in light–dark conditions provided sufficient antibacterial activity from the sixth day of cultivation onwards (Table 1). As we showed in Figure 2, this extract contained peptaibols. For *S. aureus* CCM3953, the extract from the third day of cultivation, light–dark regime suppressed the growth of bacterium to 64.4% compared to the control. The percentage of growth by testing extracts isolated on the 10th or 12th day of cultivation (light–dark) was 30.0% and 28.7%, respectively, compared to the control. The extracts isolated from the culture of *T. atroviride* O1 cultivated in constant darkness showed a similar trend in antibacterial activity. However, the efficiency was lower, and measurable antibacterial activity was detected in the extracts isolated starting from the eighth day of cultivation (90% growth compared with the control). The inhibitory effect of the extracts strengthened with the extended time of cultivation up to the tenth day (Table 1). An increase in antibacterial activity in the peptaibol-containing extracts obtained from later stages of *T. atroviride* O1 cultivation may indicate the rise in the amount of peptaibols and possibly other peptide substances in the extracts, i.e., stimulating the synergic effect in the extracts. The increase in inhibitory activity of extracts well correlated with the transcription of the gene *tex1_Ta*. The activity was detected in and after the peak of *tex1_Ta* expression, suggesting the accumulation of peptaibols in the culture of *T. atroviride* O1 (see Discussion).

To examine the possibility of modulating the antibacterial activity of extracts, the Mueller Hinton Broth (MHB) was supplemented with lipoteichoic acid (LTA, 1 mg/mL) or horse blood serum (1%). The results for MRSA are summarized in Table 1. The presence of exogenous LTA eliminated the antibacterial effect of peptaibol-containing extract from the 12th day of cultivation. The percentage of growth even increased compared with the control. The horse blood serum only slightly suppressed the inhibitory activity of the peptaibol-containing extracts. Generally, the growth of MRSA in MHB with active peptaibol-containing extracts reached between 0.4 and 4.8% compared to the control, but when MHB was supplemented with horse blood serum, the growth increased to a level ranging from 10.2 to 18.0%. These results indicate that the antibacterial effect of peptaibol-containing extracts depends on not only the cell wall composition of target microorganisms, but also on the conditions of the environment where these extracts are applied.

### 2.4. Anticancer Activity of Peptaibols in 2D and 3D Conditions

**Figure 6:**
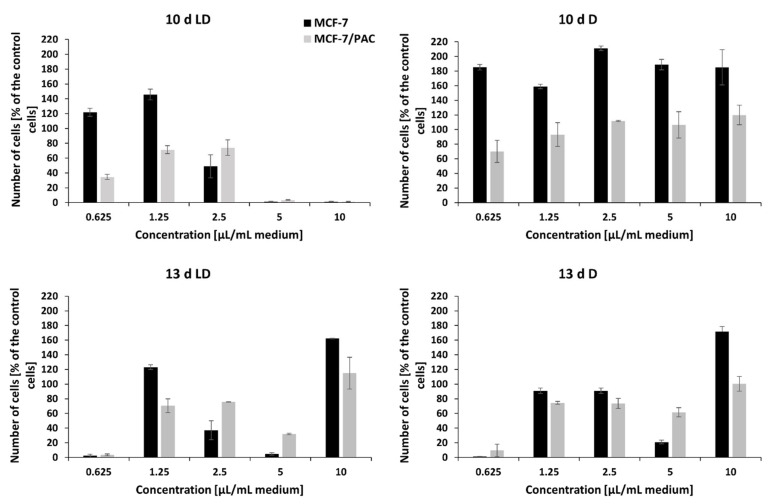
The effect of the crude extract of peptaibols on viability of human breast cancer cell line (MCF-7) and its multidrug-resistant subline (MCF-7/PAC), growing in monolayers. Tested peptaibol-containing extracts were obtained from a colony of *T. atroviride* O1 on the 10th or 13th day of cultivation (10 d, 13 d, d = day) in dark mode (D) or in a circadian rhythm (LD) on PDA agar; the amount of applied extract was 0.625, 1.25, 2.5, 5, and 10 µL per 1 mL of cultivation medium (10 d LD = 5.5, 13.1, 21.9, 43.7, and 87.5 µg/mL, respectively; 10 d D = 7.4, 17.8, 29.7, 59.4, and 118.8 µg/mL, respectively; 13 d LD = 5.2, 12.6, 21.0, 41.9, and 83.8 µg/mL, respectively; 13 d D = 6.8, 16.2, 27.0, 54.1, and 108.1 µg/mL, respectively), and the time of exposure to compounds was 72 h.

**Figure 7:**
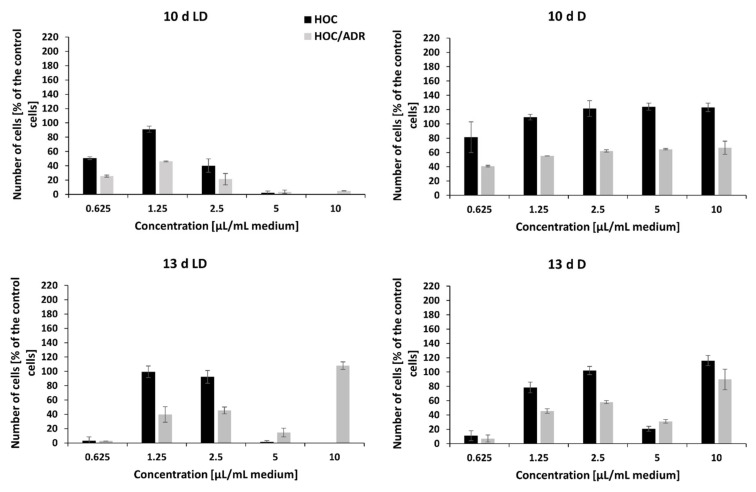
The effect of crude extract of peptaibols on viability of human ovarian cancer cell line (HOC) and its multidrug-resistant subline (HOC/ADR) growing in monolayers. Tested peptaibol-containing extracts were obtained from a colony of *T. atroviride* O1 on the 10th or 13th day of cultivation (10 d, 13 d, d = day) in dark mode (D) or in a circadian rhythm (LD) on PDA agar; the amount of applied extract was 0.625, 1.25, 2.5, 5, and 10 µL per 1 mL of cultivation medium (10 d LD = 5.5, 13.1, 21.9, 43.7, and 87.5 µg/mL, respectively; 10 d D = 7.4, 17.8, 29.7, 59.4, and 118.8 µg/mL, respectively; 13 d LD = 5.2, 12.6, 21.0, 41.9, and 83.8 µg/mL, respectively; 13 d D = 6.8, 16.2, 27.0, 54.1, and 108.1 µg/mL, respectively), and the time of exposure to compounds was 72 h.

The crude extracts of peptaibols obtained on the 3rd, 6th, and 8th day of the cultivation of *T. atroviride* O1 on PDA agar, in the dark (D) or during a light–dark (circadian) rhythm (LD) in the tested amounts (0.625 µL/mL, 1.5 µL/mL, 2.5 µL/mL, 5 µL/mL, and 10 µL/mL of cultivation medium) and concentrations (5–150 µg/mL) had no effect on the proliferation of human breast carcinoma (MCF-7) and human ovarian carcinoma (HOC) cell lines or their multidrug-resistant sublines MCF-7/PAC and HOC/ADR, respectively. However, these cell lines were inhibited with the extracts obtained on the 10th and 13th day of cultivation. In general, the effect of peptaibol-containing extracts was more signi-ficant against multidrug-resistant cells than against the sensitive ones growing in monolayers (Figure 6 and Figure 7). In the case of extracts obtained from the 10th day of cultivation, light–dark (circadian) regime (10 d LD), we detected cytotoxic activity of a dosage-dependent nature from seemingly metabolic active cells through the start of a cytotoxic effect by 2.5 µL/mL (volume of extract per 1 mL of medium = 21.9 µg/mL) to metabolic inactive cells by 5 µL/mL (43.7 µg/mL), which continued by 10 µL/mL (87.5 µg/mL)—Figure 6 and Figure 7 for drug-sensitive cell lines as well as drug-resistant sublines. Conversely, it seems possible that by extracts obtained from cultivation in the dark (10 d D) and extracts from the 13th day of cultivation (13 d LD and 13 d D), the applied amounts/concentrations were insufficient for detecting a linear cytotoxic effect. Because extracts from the 10th day of cultivation (in light–dark conditions) showed the strongest toxic effect, we were able to determine IC50 values: HOC = 2.1 µL/mL (volume of peptaibol extract per 1 mL of cultivation medium = 18.4 µg/mL), HOC/ADR < 0.625 µL/mL (< 5.5 µg/mL), MCF-7 = 2.5 µL/mL (21.9 µg/mL), and MCF-7/PAC = 2.8 µL/mL (24.5 µg/mL). Interestingly, the extracts from *T. atroviride* O1 cultivated in the dark displayed the opposite trend, i.e., inducing cell proliferation at the tested concentrations. The most significant proliferation increase was exerted by extracts obtained from the tenth day of cultivation in the dark on MCF-7 cells.

Because the most significant antiproliferative effect showed peptaibol-containing extract isolated within the 10th day of the light–dark regime of cultivation (10 d LD), its effect was also tested on spheroids (together with extracts obtained from cultivation in the dark). The trend of the antiproliferative effect against MCF-7 and HOC spheroids was comparable with that observed on 2D models of the same cell lines, i.e., extracts from the 10 d LD regime showed dosage-dependency, while the 10 d D regime extract seemingly did not.

**Figure 8:**
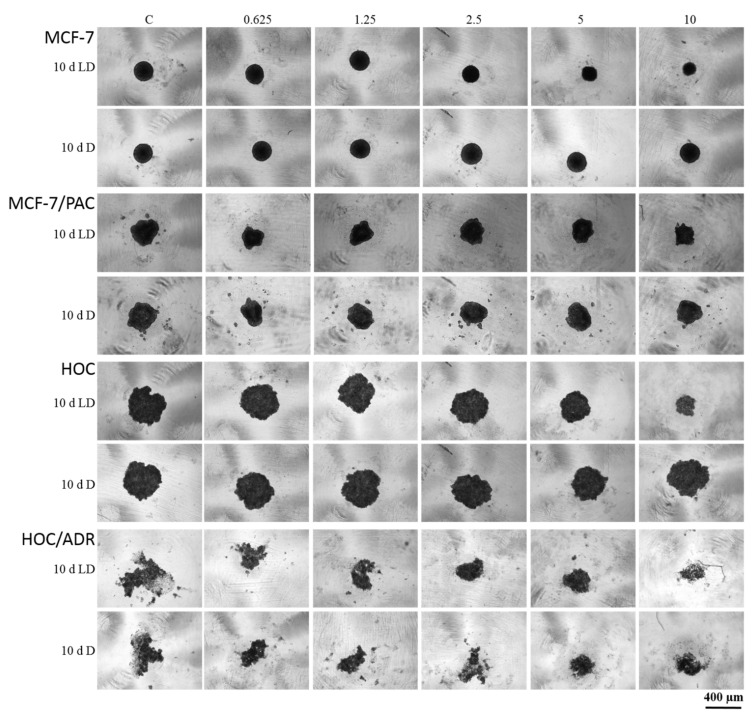
The effect of peptaibol-containing extracts on the formation of spheroids. Tested cells were human breast cancer sensitive (MCF-7) and multidrug-resistant (MCF-7/PAC) cells, human ovarian cancer sensitive (HOC) and multidrug-resistant (HOC/ADR) cells. Tested peptaibol-containing extracts were obtained from a colony of *T. atroviride* O1 on the 10th day of cultivation (10 d = day) in dark mode (D) or in a circadian rhythm (LD) on PDA agar; the amount of applied extracts was 0.625, 1.25, 2.5, 5, and 10 µL per 1 mL of cultivation medium (10 d LD = 5.5, 13.1, 21.9, 43.7, and 87.5 µg/mL, respectively; 10 d D = 7.4, 17.8, 29.7, 59.4, and 118.8 µg/mL), the time of compound action was 72 h, and a Zeiss Axio Verte.A1, Axiocam Icc 1 was the microscope used.

The sensitive cell lines (MCF-7 and HOC) formed more compact spheroids compared to multidrug-resistant cell lines (MCF-7/PAC and HOC/ADR), which formed only loose non-compact spheroids. Therefore, it is not possible to responsibly compare the effects on sensitive and multidrug-resistant cells. However, there are clearly visible differences in the size and compactness of the spheroids of all tested cell lines when cultivated in the presence of peptaibol-containing extracts from the 10th day of cultivation in the light–dark regime in an amount of 10 µL/mL (87.5 µg/mL) of incubation medium compared to the non-affected spheroids. Affected MCF-7 and HOC spheroids were significantly smaller, while the spheroids of resistant sublines were more (HOC/ADR) or less (MCF-7/PAC) disintegrated compared to their respective controls (Figure 8). The extract obtained on the 10th day of cultivation from the mycelium of *T. atroviride* O1 cultivated in constant darkness caused no noticeable changes (no effect).

## 3. Discussion

Recent years have shown the urgent need for alternatives to commonly used anti-biotics and chemotherapeutics, due to the rising levels of resistance in microorganisms [4] and cancer cells [26]. Biocontrol fungi *Trichoderma* sp. are a rich source of peptide meta-bolites called peptaibols, which are members of peptaibiotics, where cyclic peptaibiotics, lipopeptaibols, lipoaminopeptides, and other peptaibiotics also belong [17]. Peptaibols are dominant in *Trichoderma* sp., while other types of peptaibiotics are produced mainly by members of kingdom Fungi outside the genus *Trichoderma* [10]. The current research attempts to find new chemical structures capable of overcoming the recently rising phenomenon of resistance. However, the revision of peptaibols with known structures might bring just as surprising discoveries. For example, there is a recently published study regarding prealamethicins, where the authors concede the possibility of fragmented alamethicin. The cytotoxic activity was tested, though not on multidrug-resistant cell lines [29]. In this study, we report the peptaibol-containing extracts obtained from the *Trichoderma atroviride* O1 strain in the context of fighting microbial and cancer cell resistance. The detection of peptaibols was based on MALDI-TOF mass spectrometry analysis (Figure 1). The *m/z* profile refers to atroviridins, peptaibols described for *Hypocrea atroviridis* (teleomorph = sexual reproductive stage of *Trichoderma* *atroviride*). Compared to the most studied and commercially available peptaibol alamethicin (Sigma-Aldrich, St. Louis, MO, USA) Ac-Aib-Pro-Aib-Ala-Aib-Ala-Gln-Aib-Val-Aib-Gly-Leu-Aib-Pro-Val-Aib-Aib-Glu-Gln-Phol [18], atroviridins can be regarded as alamethicin analogues. Generally, peptaibols are naturally produced as a mixture of peptides differing in some amino acid positions (changes between Aib, Ala, Isoleucine/L, and Isovaline/V) [10]. Based on the fact that antibacterial and cytotoxic activity was described for alamethicin [16], a similar effect of peptaibols of *T. atroviride* O1 can be expected. Moreover, we detected other compounds of a peptide nature in the extracts. For the low molecular mass peaks *m/z* ~ 800 and *m/z* ~ 1000, we cannot exclude the presence of yet undescribed peptaibols (typical mass 500–2100 Da), but the peaks appear very early in cultivation contrary to the detected peaks of atroviridines (though different *m/z* had their highest intensities at different times of cultivation). In the early stages of cultivation, no biological activity was detected (Section 2.3 and Section 2.4). Molecules represented by *m/z* ~ 2140 and 2370–2390 exceeded the molecular mass of peptaibols (2100 Da). Due to the nature of extracts used in this work—crude extracts of peptaibols—the prospect of other natural compounds might be a possibility. By assuming their peptide character (we used an extraction method designed to obtain peptaibols = peptides for MALDI-TOF analysis [30]), there is a probability of contribution to synergic effect with isolated peptaibols (though other effects are also possible by cytotoxic activity—Discussion, final paragraph). Conversely, *Trichoderma* sp. has been studied since the 1930s, so the biologically active compounds (of peptide nature) besides peptaibols would probably be partly known. Synergic effect is also the case for the peptaibols themselves as they are produced as a mixture and isolated as a mixture of peptides differing in amino acid at a few positions [8,31].

By analyzing mass spectra of the crude extracts of peptaibols, we found that light stimulates the production of peptaibols *T. atroviride* O1, which is in accordance with results described for *H. atroviridis* [28], where the synthesis of peptaibols was ascribed to conidiation, which is stimulated by blue light. However, we also detected the presence of peptaibols in extracts from cultures of *T. atroviride* O1 cultivated in constant darkness. The production was significantly delayed compared to light–dark conditions. This is in contrast with the work of Komon-Zelazowska et al., (2007), who did not detect peptaibols in cultures cultivated in darkness. The production of peptaibols is often strain-specific [11]. Therefore, environmental isolate such as *T. atroviride* O1 [27] could have enhanced the secondary metabolites production (including peptaibols) compared to *H. atroviridis* ATCC 74058 (=P1) and IMI 206040, which could make it an interesting production strain of 20-residue peptaibols. Another possible explanation was provided by Komon-Zelazowska et al., (2007), who detected peptaibols under the conditions of starvation regardless of light conditions [28]. *T. atroviride* O1 was cultivated in 25 mL of PDA medium, so after five days of cultivation, the amount of nutrients in medium could have been limited, causing the fungus to trigger the production of peptaibols.

To analyze peptaibol production at the level of RNA, we examined the expression profile of peptaibol synthetase *T. atroviride* O1 encoded by the gene *tex1_Ta* [23,28]. The production of peptaibols by peptaibol synthetases (non-ribosomal peptide synthetases) is a known fact [11]. We wanted to explain the observation of the assessment of biological activity (“the later in cultivation, the more effective extract”). When cultivated in light–dark conditions, the *tex1_Ta* transcript was detectable from the third day of cultivation (Figure 4), which was the same time the peptaibols reached a level detectable by MALDI-TOF mass spectrometry. In the constant darkness, the expression of the peptaibol synthetase exceeded the detection limit on the fifth day of cultivation. However, peptaibols were detectable on the mass spectrum one day later, on the sixth day. The expression profile of *tex1_Ta* in constant darkness (Figure 5) shows that the reference (12th day) has a very low expression of peptaibol synthetase. The expression on the fifth day of cultivation was only 2-fold higher compared with the reference. On the sixth day of cultivation, when peptaibols are detected, the *tex1_Ta* expression is 12-fold that of the reference. In comparison, the expression level on the third day of cultivation in light–dark conditions is above 5-fold compared with the reference (Figure 4). Both cases suggest that a much higher level of gene expression of peptaibol synthetase was needed for peptaibols to be produced. These results suggest that the production of detected peptaibols (atroviridins) is indeed catalyzed by the product of the gene *tex1_Ta* and, as we showed in Section 2.3 and Section 2.4, the biological activity in the extracts appears predominantly in and after the peak of expression of *tex1_Ta*, which indicates peptaibols are sufficiently produced in a mixture of metabolites, showing their synergism. Moreover, the stage at which secondary metabolites (where peptaibols belong) are synthetized is the late stationary phase of growth [32]. This explains why the peak of *tex1_Ta* expression is on the tenth or ninth day of cultivation for light–dark conditions and constant darkness, respectively. Because the natural environment for *Trichoderma* sp. is the rhizosphere, where no light is present [8], we hypothesize that the growth processes of *T. atroviride* O1 are faster in the dark, so the peak of secondary metabolite production is reached faster.

Alamethicins were described to have antibacterial activity against Gram-positive bacteria [16]. We expected the presence of atroviridins (alamethicin analogues) in peptaibol-containing extracts, so the collection strain of *Staphylococcus aureus* CCM 3953 and clinical isolate of methicillin resistant *Staphylococcus aureus* (MRSA) were used in this study as model bacteria to differentiate antimicrobial activity against sensitive and resistant bacterial strains. Indeed, antibacterial activity was detected (Table 1) with a more pronounced effect on MRSA. The inhibitory activity of extracts depended on the applied extract. Al-though peptaibols were present in the extract of *T. atroviride* O1 cultivated for three days in light–dark conditions, the effect on the growth of both *S. aureus* was not detected. This suggests that the effective concentration of peptaibols was not reached in this extract. However, the extracts isolated from later stages had antibacterial activity. This is also the case for the extracts isolated from *T. atroviride* O1 cultivated in constant darkness. Despite peptaibols being detected by MALDI-TOF analysis in the extract from the sixth day of cultivation, there was no detectable antibacterial activity. Longer cultivation was needed to cause the antibacterial effect. Although a rapid decrease in *tex1_Ta* transcription on the 12th day of cultivation (regardless of light condition) was observed, the peptaibol-containing extracts had strong antibacterial activity (Table 1). So even with the possible degradation of the peptaibols, their amount present in the extracts remained above the effective dose for the inhibition of Gram-positive bacteria. Taken together, we report that peptaibol-containing extracts from *T. atroviride* O1 have potential to inhibit the growth of Gram-positive bacteria, including methicillin-resistant *S. aureus*.

The mechanisms that lower the antibacterial activity of cationic peptides have been reviewed [1,5]. We attempted to clarify the mechanism of action used by neutral peptides (to which peptaibols belong). Peptaibols have low antibacterial activity against Gram-ne-gative bacteria due to the interaction lipopolysaccharide (LPS)—peptaibol [16]. We hypothesized a similar interaction in Gram-positive bacteria, i.e., an interaction of lipoteichoic acid (cell wall component of Gram-positive bacteria) with peptaibols. Therefore, we measured the combined effect of a crude extract of peptaibols (obtained in the 12th day of cultivation) mixed with lipoteichoic acid (LTA) from *S. aureus* on the growth of methicillin-resistant *S. aureus*. The activity of peptaibol-containing extracts was hampered in the presence of LTA. Cationic antimicrobial peptides bind to LTA, and the microorga-nism can evolve resistance to this type of peptides upon chemical modification of the LTA backbone (reduction in the overall negativity of bacterial membrane) [5,33]. Though atroviridins, analogues of alamenthicins, are neutral peptides, they also seem likely to bind to LTA. Though the interaction with other components of extracts cannot be refuted, there is an indication of interaction LTA-peptaibol, which to our best knowledge has not yet been described. It may be possible that LTA facilitates transport of peptaibols (and cationic peptides) by directing them through the cell wall of Gram-positive bacteria to the plasma membrane. Because neutral peptides interact with the plasma membranes of microorganisms by hydrophobic interaction, it is unclear whether changes in the LTA structure can evolve, causing resistance similar to cationic peptides. We also observed that the percentage of growth in the presence of LTA and peptaibol-containing extract was higher than that of the control. LTA is a key element of the biofilm of *S. aureus* [34]. To protect from peptaibols, *S. aureus* may form a biofilm, also using available LTA. During our experiment, we observed/visually detected features of biofilm forming at the bottom of the cultivation vessel containing LTA and the crude extract of peptaibols. Because the growth parameter we measured was absorbance at 630 nm, the biofilm at the bottom of the well in the microplates would lead to increased absorption compared to the cell suspension (control). Alternatively, it could be a lower amount of peptaibols in the extracts, which act as a signal for cells of *S. aureus* to group and form biofilm as a rescue mechanism. This would explain why higher growth (%) than in the control was also observed in samples of tested extracts, where no antibacterial activity was detected.

We tested whether the presence of blood serum had an impact on the inhibition of the growth of methicillin resistant *S. aureus* (MRSA) mediated by peptaibol-containing extracts. We opted for horse blood serum in our experiments. The studies with implications for veterinary medicine may be perceived slightly less groundbreaking compared with discoveries with an impact on human medicine. However, MRSA is a serious threat for horses (*Equus* sp.) and has already established possible routes of transmissions to humans (for now, fortunately, not so frequently exploited) [35,36]. The proteolytic activity of serum proteins can be regarded as a major concern. However, α-aminoisobutyric acid of peptaibols is a quaternary amino acid that, due to its structure, hinders peptidase attack [37]. We found that horse blood serum had a low influence on the antibacterial activity of peptaibol extracts. Extracts with the strongest inhibitory activity against MRSA (8th, 10th, and 12th day of both types of light regimes) reduced the growth of the bacterium under 5.0% compared with the control, whereas, in the medium with horse serum supplement, the percentage of growth was between 10.2% and 18.0% compared to the control, thus the activity of peptaibol-containing extracts was only mildly hampered. This suggests that the peptidase activity of serum may not be completely blocked by the presence of α-aminoisobutyric acid in the structure of peptaibiotics. The above-mentioned findings suggest that the activity of peptaibols of *T. atroviride* O1 depends on the cell wall composition of target bacteria as well as on the environment where it is applied.

The mechanism of action of (neutral) peptaibols on cancer cells viability and proli-feration remains to be elucidated. However, the potential of cationic antimicrobial peptides (AMPs) as anticancer agents has been reviewed [38,39] and new candidate peptides are still emerging [40]. Only a few publications deal with the antitumor effects of peptaibols. Shi et al., observed that trichokonin VI peptaibol from *Trichoderma pseudoko-ningii* SMF2 suppressed the viability of hepatocellular cancer cells in a dose-dependent manner [26]. Rivera-Chávez et al., (2017) analyzed the cytotoxic activity of prealamethicin F50 and related peptaibols against a panel of seven cell lines, with the activity in the single digit µM range [29]. Kavianinia et al., assessed culicinin D, a peptaibol/lipoaminopeptide originally isolated from *Culicinomyces clavisporus*, and its analogues for antiproliferative activity against three breast cancer cell lines (MDA-MD-468, SKBR3, and T47D) as well as against a non-small cell lung cancer cell line, NCI-H460. This metabolite exhibited antiproliferative activity with IC50 values mostly in the concentration range of 0.001–0.35 µM [41]. Discernibly for this work, we tested the effect of a crude extract of peptaibols from *T. atroviride* O1 on a 2D model of breast cancer and human ovarian cell lines and their paclitaxel- and doxorubicin-resistant sub-lines, respectively. Comparably to antimicrobial activity, the cell lines were affected when treated with extracts of peptaibols obtained from later stages of cultivation (10th, mainly, and 13th day), suggesting the effect of accumulation of peptaibols, particularly on the tenth day, which is also a peak day for expression of peptaibol synthetase *tex1_Ta* in the light–dark regime (LD) of cultivation (Figure 4). Although we observed the differences in size and compactness of spheroids, no unamb-iguous conclusion regarding the significance of the effect of peptaibols between anticancer drug-sensitive and resistant cell lines can be drawn.

However, there are some aspects of the results that need to be addressed. Throughout the study, there were observed differences in an effect of peptaibol-containing extracts on sensitive and drug resistant cell lines. In the 2D model, the crude extracts of peptaibols exerted stronger antiproliferative activity against MCF-7/PAC and HOC/ADR compared to their sensitive counterparts (Figure 6 and Figure 7). Although AMPs, and therefore anticancer peptides (ACPs), can act in a non-lytic manner on target cells [38], peptaibols (including the most studied alamethicin) are regarded as plasma membrane targeting molecules [18]. Compared to healthy cells, cancer cells may have an altered amount of cholesterol in the membrane, and another hallmark is an exposure of negatively charged phosphatidylserine on the outer leaflet of the membrane (which facilitates binding of cationic peptides as ACPs) [42,43]. However, multidrug-resistant cancer cell lines have increased plasma membrane rigidity compared to sensitive cancer cells, mainly due to the even higher amounts of cholesterol present in the membrane [44]. In doxorubicin-resistant breast cancer cell line MCF-7/ADR, this was accompanied by increased levels of cholesterol esters, phosphatidylinositol, and sphingomyelin in the membrane [45], resulting in a more rigid membrane, which together with a P-glycoprotein efflux pump prevented doxorubicin import [46]. Cholesterol and sphingomyelin are normally building blocks of lipid rafts, which are rigid structures in an otherwise fluid plasma membrane. However, apart from the mentioned changes in cancer cells, the rigidity may also increase by the higher amount of saturated fatty acids present in the membrane [42], which was also detected in breast cancer tissue [47]. This results in an increase in lipid packing density, which was proven on model membranes to act as a physical barrier for paclitaxel intake [48]. Moreover, the peptide azurin increased plasma membrane permeability of the MCF-7 cells, sensitizing them to doxorubicin as well as paclitaxel [49]. Notably, lipid composition of the plasma membrane is what distinguishes MRSA from susceptible *S. aureus*. MRSA has a more rigid membrane attributed to a different fatty acid composition [50]. The contribution of *cls* (cardiolipin synthase) and *psgA* (phosphatidylglycerol synthase) is also suggested [51]. This leads to the possibility of collateral sensitivity, a phenomenon where evolved resistance to one drug increases sensitivity to a second drug [52]. In this case, to the peptaibols (they may need a more rigid membrane to exert inhibitory activity). However, this hypothesis requires further extensive study.

The extract obtained from the dark cultivation increased proliferation of the tested cell lines, especially the MCF-7 cell line (Figure 6). The drawback of using crude extracts may lie in the presence of other substances besides the desired metabolites, as we confirmed by MALDI-TOF analysis (Figure 2 and Figure 3). Some of these substances could have caused metabolic changes to cancer cells, thus hampering the effect of peptaibols. However, peptaibols in the extract could still be somewhat active because the growth of a drug-resistant subline reached a level comparable to the control (Figure 6), while the anticancer-drug-sensitive MCF-7 cell line under the influence of peptaibol-containing extracts obtained from cultivation in the dark (10th day) showed a standing just below 200% compared to the control (Figure 6). An effect of a similar nature could also have been the case for the extract from the 13th day of cultivation in the light–dark regime. Because of applied amounts below linear cytotoxic activity, the other substances could exert in 10 µL/mL (83.8 µg/mL) a stimulatory effect on cell lines, while in different amounts, there is an antiproliferative effect. (The effect of some constituents in the extracts might not be only synergic, but also, in just the right applied amount, the stimulatory effect of a constituent prevails over the antiproliferative of another one and vice versa). The effect of peptaibol-contai-ning extracts at the molecular level will be investigated in a future study. To sum up, our results, together with known information regarding the effect of peptaibols on cancer cells show that these metabolites are potential candidates in the field of antimicrobial and anti-cancer peptides.

## 4. Materials and Methods

### 4.1. Microbial Strains and Cultivation Conditions

*Trichoderma atroviride* strain O1 [27] was used throughout this study. For pre-cultivation, the filamentous fungus was pre-grown for three days on potato dextrose agar (PDA; Sigma-Aldrich, St. Louis, MO, USA) plates at 21 °C (IKA KS 400 ic control, Staufen, Germany). An agar plug (5 mm) of the actively growing colony margin was then propagated twice, after two days each, inoculated to the center of a fresh PDA plate, and cultivated in light–dark conditions (circadian rhythm, daylight ~10 h, November, Central Europe, 2020) or in complete darkness (21 °C).

Gram-positive bacteria *Staphylococcus aureus* CCM3953 (Czech Collection of Microorganisms, Masaryk University, Brno, Czech Republic) and methicillin resistant *Staphylococcus aureus* (MRSA; clinical isolate from a central venous catheter, resistant against penicillin, methicillin, cefoxitin, erythromycin, chloramphenicol, and ciprofloxacin; MecA gene confirmed) were used for determination of the antimicrobial activity of isolated peptaibols. The bacteria were stored at −80 °C in glycerol and inoculated on fresh Mueller Hinton Agar (MHA, Biolife, Milan, Italy) two days prior to the experiment and incubated at 37 °C for 24 h.

### 4.2. Isolation of Peptaibols

An agar plug (5 mm) of propagated *T. atroviride* O1 was inoculated on a fresh PDA medium (25 mL in a Petri dish with diameter 10 cm) covered with cellophane. The culture was incubated at 21 °C for 1, 2, 3, 4, 5, 6, 8, 10, 12, and 13 days of cultivation (light–dark or constant darkness), followed by the isolation of peptaibols as described previously [30]. Briefly, the actively growing colony margin was collected and homogenized by liquid nitrogen. A total of 0.15 g of mycelium powder was subjected to extraction by 1 mL of 60% ethanol (Mikrochem, Pezinok, Slovakia). The extraction procedure consisted of three times repeated 1 min vortexing followed by 5 min incubation at laboratory temperature. The last step was centrifugation for 5 min at 19,000× *g*. The supernatant containing peptaibols was transferred into a new vial and stored at −20 °C until analyzed. The extracts isolated on 3rd, 6th, 8th, 10th, 12th, or 13th day of cultivation were applied by biological activity testing. A starting material of 0.3 g of mycelium powder (mycelium of fungus crushed in liquid nitrogen) was used to obtain peptaibol-containing extracts, which were tested for antibacterial and cytotoxic activity.

After the extraction, the solvent (60% ethanol) was removed from the aliquot volume of peptaibol-containing extracts and placed in pre-weighed microtubes by vacuum evaporation using Eppendorf Concentrator plus (Eppendorf, Hamburg, Germany). Once solid pellet was reached, the weight of the microtubes was determined by analytical balances (Satorius, Göttingen, Germany), hence we express the concentration of peptaibol-containing extracts used in the next experiments in units of µg/mL.

### 4.3. MALDI-TOF Analysis of Low-Molecular Mass Peptides

The analysis of obtained extracts by Matrix-Associated-Laser-Desorption-Ionization with Time-of-Flight detector was performed as described previously [30]. A total of 1 µL of the extract was mixed with 1 µL of matrix solution (10 mg of 2.5-dihydroxybenzoic acid mL^−1^ (Bruker Daltonics, Billerica, MA, USA) in acetonitrile/methanol/water (1:1:1, *v/v/v*) and 0.3 % trifluoroacetic acid (Sigma-Aldrich, St. Louis, MO, USA)). Then, 1 μL of the sample was directly spotted onto the target plate (MTP 384 plate ground steel BC, Bruker Daltonics, Billerica, MA, USA) and allowed to dry before analysis.

Measurements were performed in a reflector positive mode. A Peptide calibration standard II (Bruker Daltonics, Billerica, MA, USA) was used for calibration with *m/z* between 700 and 3500 Da. Mass spectra were compared with the available literature of peptaibols [28,30]. Only very strong differences in the mass spectra (present versus not present) were considered relevant and used for the interpretation of results.

### 4.4. Transcriptional Analysis of Gene tex1_Ta Encoding Peptaibol Synthetase T. atroviride O1 by Real-Time PCR

An agar plug of propagated *T. atroviride* O1 was inoculated on fresh PDA medium covered with cellophane. The culture was incubated at 21 °C and cultivated in light-dark conditions or constant darkness for 1, 2, 3, 4, 5, 6, 8, 9, 10, 11, and 12 days. Total RNA was isolated using TRI reagent (Sigma-Aldrich, St. Louis, USA) from the harvested actively growing colony margin homogenized by liquid nitrogen. Isolated RNA was treated with DNase I (Sigma-Aldrich, St. Louis, USA) and reverse transcribed with the AllScript Reverse Transcriptase 4 U/µL (Biotechrabbit, Berlin, Germany) with oligodT16 (Metabion international AG, Planegg, Germany). qPCR was performed with 4× CAPITAL qPCR Green Master Mix (Biotechrabbit, Berlin, Germany) and CFX Connect Real-Time System (Bio-Rad, Hercules, California, USA) and the primers:

- *tex1_Ta* fwd: 5′GGTACACGTCTCTGCCGCTATGC and
- *tex1_Ta* rev: 5′CATTTCGGTGCCAGCGTACGCGG [23].

Expression ratios were calculated by 2^−ΔΔCt^ method [53] using *sar1* as a reference gene:

- *sar1* fwd: 5′CTCGACAATGCCGGAAAGACCA,
- *sar1* rev: 5′TTGCCAAGGATGACAAAGGGG [54].

Analysis was performed on biological duplicates and technical triplicates. The significance of the differences between sample and control were evaluated by an unpaired two-tailed Student’s *t*-test, and a *p*-value of less than 0.05 was considered significant.

### 4.5. Antibacterial Activity of Peptaibols of T. atroviride O1

The antibacterial activity of the isolated peptaibols was determined by the micro-dilution method in Mueller Hinton Broth (MHB, Biolife, Milan, Italy). An overnight culture of bacteria (cultivated in MHB at 37 °C, 400 rpm) was prepared from the fresh culture on MHA. The overnight inoculum served for inoculation of fresh MHB (1% inoculum). Microorganisms were cultivated in a microplate at 37 °C under shaking (400 rpm). One well of the plate contained 190 µL of inoculated MHB and 10 µL of the crude extract of peptaibols (i.e., 50 µL of extract in 1 mL of medium). The final concentration of peptaibol-containing extracts obtained from light-dark (circadian) conditions was: 3rd day = 362.5 µg/mL, 6th day = 450.0 µg/mL, 8th day = 750.0 µg/mL, 10th day = 437.5 µg/mL, 12th day = 430.5 µg/mL. For the extracts obtained from the cultivation in dark: 3rd day = 255.0 µg/mL, 6th day = 425.0 µg/mL, 8th day = 750.0 µg/mL, 10th day = 593.8 µg/mL, 12th day = 571.4 µg/mL. For the control, 10 µL (50 µL/mL of medium) of 60% ethanol was used. Absorbance at 630 nm (A_630_) was measured as the growth parameter. The measurement was performed until microorganisms reached the stationary phase of growth. The effect of peptaibol-containing extracts on the cultivation of bacteria was evaluated using percentage of growth, i.e., the A_630_ of the sample divided by the A_630_ of the control multiplied by 100. Two independent experiments were performed with samples in triplicate. The interaction of peptaibols with lipoteichoic acid (LTA) was evaluated after supplementation of MHB with 1 mg/mL lipoteichoic acid from *S. aureus* (Sigma-Aldrich, St. Louis, MO, USA). For an evaluation of the effectiveness of peptaibols-containing extracts, MHB was supplemented with 1% horse blood serum (Oxoid Limited, Hampshire, UK).

### 4.6. In Vitro Test of Antiproliferative Effect of Crude Extracts of Peptaibols in 2D and 3D Conditions

Human ovarian cancer cell line HOC (A2780, Sigma-Aldrich), human breast cancer cell line MCF-7 (kindly provided by Professor Ján Kovář, Third Faculty of Medicine, Charles University, Prague), and their multidrug resistant sub-lines HOC/ADR (A2780ADR, Sigma-Aldrich) resistant to doxorubicin and MCF-7/PAC [55,56] resistant to paclitaxel were cultivated in DMEM (Sigma-Aldrich) medium supplemented with 10% FBS and 1% antibiotic antimycotic solution (Sigma-Aldrich). Doxorubicin at the final concentration of 0.08 µM or paclitaxel at the final concentration of 0.3 µM were added to the culture medium to maintain the resistance of the cell lines. All cell lines were maintained in a humidified atmosphere containing 5% CO2 at 37 °C.

The cells growing in a monolayer were seeded at a concentration of 1 × 10^5^ cells in 1 mL medium per well of a 96-well plate and incubated for 24 h. Afterwards, the cells were washed with PBS, and 100 µL fresh DMEM medium with 0.0625, 0.125, 0.25, 0.5, and 1 µL of the tested extracts of peptaibols was added to each well, except for the control wells. Cells were cultivated for 72 h. After incubation, the resazurin assay was performed. The cells were incubated with resazurin (0.03 mg/mL in PBS) for 2 h, after which the fluorescence was recorded (ex./em. 560/590 nm).

The U-shaped surface of 96-wells was covered with a microlayer of SeaKem LE Agarose (Lonza, Switzerland) enabling cultivation of cells in the form of spheroids. The number of 1 × 10^4^ cells was used for the inoculation of the individual wells of the adjusted U-shaped 96-well plates. Tested crude extracts of peptaibols in the amounts of 0.625, 1.25, 2.5, 5, and 10 µL/mL of cultivating medium were added 24 h after the inoculation. After the next 72 h, the effect of peptaibol extracts on the formation of spheroids was observed using a light microscope Axio Vert. A1 (Zeiss, Jena, Germany) with photo documentation equipment Axiocam ICC 1 and Axio Vision 4.8 software.

## 5. Conclusions

Peptaibols are an intriguing group of natural peptides with studied biological activity. This work deals with peptaibol-containing extracts from *Trichoderma atroviride* O1 in a contemporary approach to their application for the fight against the resistance of microorganisms and cancer cell lines. The mixture of peptaibols might manifest a synergic effect, possibly also with other constituents of extracts. The activity of gene *tex1_Ta* encoding peptaibol synthetase seemed to substantiate the observed biological activity. Surprisingly, multidrug-resistant bacterium and cancer cell lines were affected. Nevertheless, the validation/revision for the use of peptaibols *T. atroviride* O1 as antimicrobial and anticancer peptide requires further investigation on more bacterial and cancer cell models.

## Figures and Tables

**Table 1:** The growth of Gram-positive bacteria in the presence of peptaibol-containing extracts of *Trichoderma atroviride* O1 and in combination with lipoteichoic acid (LTA) or horse blood serum.

Extract ^a^	Concentration (µg/mL)	% Growth			
		*Staphylococcus aureus* CCM 3953	MRSA ^c^	MRSA (+ 1 mg/mL LTA)	MRSA (+1% Horse Serum)
Control	0 ^b^	100.0	100.0	100.0	100.0
3 d LD	362.5	150.4	100.6	- ^d^	119.1
6 d LD	450.0	64.4	3.0	-	14.5
8 d LD	750.0	34.0	2.5	-	18.0
10 d LD	437.5	30.0	1.5	-	16.7
12 d LD	430.0	28.7	0.9	143.4	16.6
3 d D	255.0	153.7	112.4	-	117.4
6 d D	425.0	143.6	107.0	-	124.7
8 d D	750.0	90.0	4.8	-	10.2
10 d D	593.8	40.6	0.4	-	15.6
12 d D	571.4	49.2	0.5	136.0	12.2

^a^ The filamentous fungus was cultivated in light–dark (LD) conditions or in complete darkness (D). Time of isolation of peptaibols: 3rd, 6th, 8th, 10th, and 12th d = day. ^b^ 50 µL/mL of 60% ethanol (solvent of extracts) was added. Extracts were also applied in the amount of 50 µL/mL. ^c^ MRSA = methicillin resistant *Staphylococcus aureus*. ^d^ - = not assayed.

## Data Availability

Not applicable.

## Supplementary Materials

The following are available online, Figure S1: MALDI-TOF/TOF (MS/MS) spectrum of fragmented ion *m/z* 1932., Figure S2: MALDI-TOF/TOF (MS/MS) spectrum of fragmented ion *m/z* 1962., Figure S3: MALDI-TOF/TOF (MS/MS) spectrum of fragmented ion *m/z* 2001.

## Sample Availability

All samples are available from the authors upon request.
