# Supplementary material for: Peptaibol-Containing Extracts of Trichoderma atroviride and the Fight against Resistant Microorganisms and Cancer Cells
Source: Molecules. 2021 Oct 4;26(19):6025. doi: 10.3390/molecules26196025 (PMC8512731; doi:10.3390/molecules26196025)
Supplement: Supplementary file 1 [file molecules-26-06025-s001.zip › molecules-1390556-supplementary.pdf]

Supplementary materials

Peptaibol-Containing Extracts of *Trichoderma atroviride* and the Fight Against Resistant Microorganisms and Cancer Cells

Ján Víglaš [1,*], Simona Dobiasová [2], Jitka Viktorová [2], Tomáš Ruml [2], Vanda Repiská [3], Petra Olejníková [1] and Helena Gbelcová [3]

[1] Institute of Biochemistry and Microbiology, Faculty of Food and Chemical Technology, Slovak University of Technology in Bratislava, 812 37 Bratislava, Slovakia; petra.olejnikova@stuba.sk
[2] Department of Biochemistry and Microbiology, University of Chemistry and Technology Prague, 166 28 Prague, Czech Republic; simka.dobiasova@gmail.com (S.D.), jitka.prokesova@vscht.cz (Jitka Viktorová), tomas.ruml@vscht.cz (T.R.)
[3] Institute of Medical Biology, Genetics and Clinical Genetics, Faculty of Medicine, Comenius University, Bratislava, Špitálska 24, 813 72 Bratislava, Slovakia; vanda.repiska@fmed.uniba.sk (V.R.), helena.gbelcova@fmed.uniba.sk (H.G.)
* Correspondence: jan.viglas@stuba.sk

Figure S1 – S3 depict fragmentation of chosen ions in the range corresponding to atroviridins/peptaibols of *Trichoderma atroviride* (Figure 1). The presence of α-amino isobutyric acid (Aib) suggest the identity of compounds as peptaibols, while also the spectra themselves bears features of MALDI-TOF/TOF measured peptaibol spectra by Brito *et al*. (2014) [21] and Katoch *et al.* (2019) [22] – increased number of peaks and partial sequences, but sufficient to provide evidence for presence of Aib – characteristic for peptaibols. Annotation of the sequence (indication of amino acids) was performed using FlexAnalysis programme (Bruker Daltonics, Billerica, Massachusetts, USA). Canonical amino acids are indicated using single-letter code, while α-amino isobutyric acid, isoleucine and isovaline are indicated as Aib, Ile and Iva, respectively.

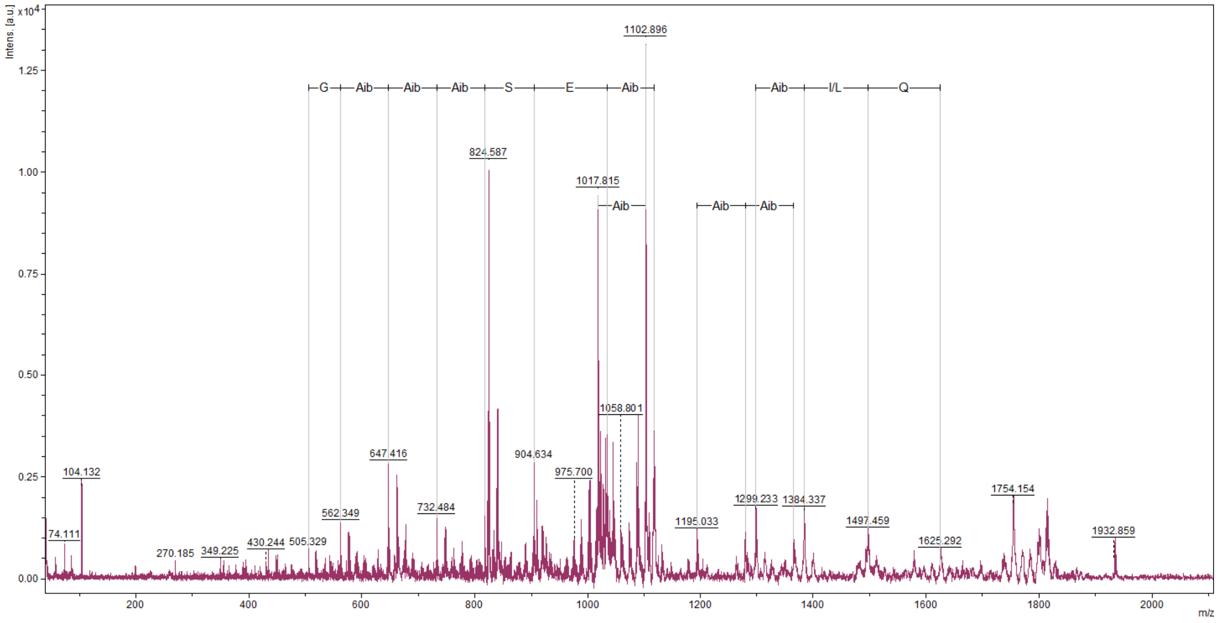

Figure S1. MALDI-TOF/TOF (MS/MS) spectrum of fragmented ion *m/z* 1932.

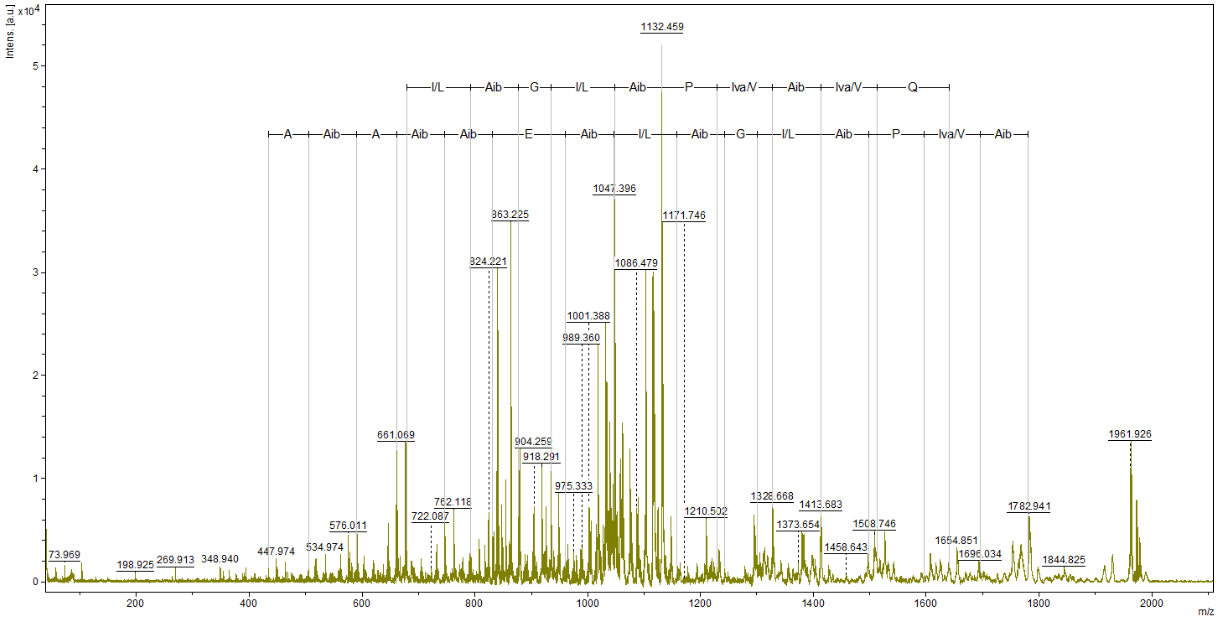

Figure S2. MALDI-TOF/TOF (MS/MS) spectrum of fragmented ion *m/z* 1962.

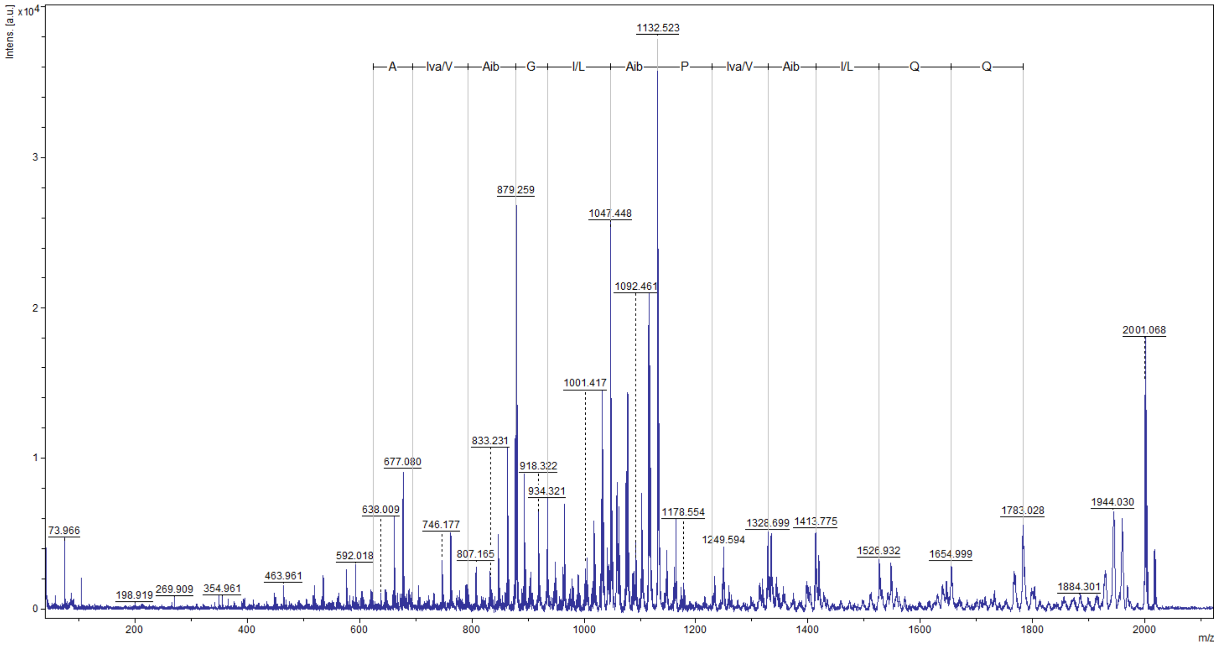

Figure S3. MALDI-TOF/TOF (MS/MS) spectrum of fragmented ion *m/z* 2001.
